# Gastroprotective Effect of Ginger Rhizome *(Zingiber officinale)* Extract: Role of Gallic Acid and Cinnamic Acid in H^+^, K^+^-ATPase/*H. pylori* Inhibition and Anti-Oxidative Mechanism

**DOI:** 10.1093/ecam/nep060

**Published:** 2011-06-23

**Authors:** Siddaraju M. Nanjundaiah, Harish Nayaka Mysore Annaiah, Shylaja M. Dharmesh

**Affiliations:** Department of Biochemistry and Nutrition, Central Food Technological Research Institute, CSIR, Mysore 570 020, Karnataka, India

## Abstract

*Zinger officinale* has been used as a traditional source against gastric disturbances from time immemorial. The ulcer-preventive properties of aqueous extract of ginger rhizome (GRAE) belonging to the family Zingiberaceae is reported in the present study. GRAE at 200 mg kg^−1^ b.w. protected up to 86% and 77% for the swim stress-/ethanol stress-induced ulcers with an ulcer index (UI) of 50 ± 4.0/46 ± 4.0, respectively, similar to that of lansoprazole (80%) at 30 mg kg^−1^ b.w. Increased H^+^, K^+^-ATPase activity and thiobarbituric acid reactive substances (TBARS) were observed in ulcer-induced rats, while GRAE fed rats showed normalized levels and GRAE also normalized depleted/amplified anti-oxidant enzymes in swim stress and ethanol stress-induced animals. Gastric mucin damage was recovered up to 77% and 74% in swim stress and ethanol stress, respectively after GRAE treatment. GRAE also inhibited the growth of *H. pylori* with MIC of 300 ± 38 **μ**g and also possessed reducing power, free radical scavenging ability with an IC_50_ of 6.8 ± 0.4 **μ**g mL^−1^ gallic acid equivalent (GAE). DNA protection up to 90% at 0.4 **μ**g was also observed. Toxicity studies indicated no lethal effects in rats fed up to 5 g kg^−1^ b.w. Compositional analysis favored by determination of the efficacy of individual phenolic acids towards their potential ulcer-preventive ability revealed that between cinnamic (50%) and gallic (46%) phenolic acids, cinnamic acid appear to contribute to better H^+^, K^+^-ATPase and *Helicobacter pylori* inhibitory activity, while gallic acid contributes significantly to anti-oxidant activity.

## 1. Introduction

More and more evidences are being accumulated nowadays regarding the cause of gastric hyperacidity and ulcers. Stress appear to play a major role as indicated by a set of studies which emphasizes that any patient irrespective of the nature of the disease, if admitted to emergency wards in the hospital, invariably ends up with gastric ulcers [1].

**Scheme 1:**
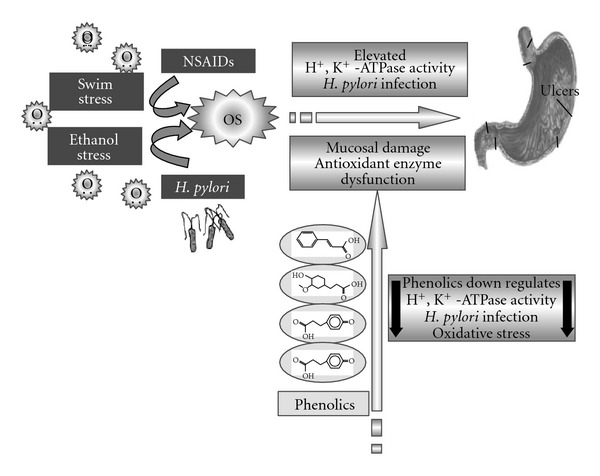
Ulcerogens generate oxidative stress (OS) leading to susceptibility for ulcer formation by activating H^+^, K^+^-ATPase, enabling *H. pylori* colonization and invasion, mucosal damage, and so forth, ginger downregulates these events.

Besides this there are characteristic problems such as (i) Zollinger-Ellisson syndrome where there is a high and uncontrolled production of acid; (ii) the use of non-steroidal anti-inflammatory drugs [2] (NSAID) for rheumatoid diseases and (iii) a rod-shaped pathogenic bacteria *Helicobacter pylori*, normally existing in human stomach are known to cause ulcers [3]. Ulceration may occur either by uncontrolled production of acid or by the side effects of NSAIDs which acts as inhibitors of gastric mucosal defense or by manipulating the mucosal epithelium structure-function causing a defenseless condition and hence ulcers. The concept of management of ulcer disease is fast changing. Treatment was based on the principle that excessive secretion of acid is the reason for ulcer symptoms. However, understanding the role of histamine, gastrin and acetylcholine in addition to gastric acid in controlling gastric secretion lead to the designing of anti-ulcer drugs which act as blockers of such receptors. The role of enzymic gastric proton pump with H^+^, K^+^-ATPase activity is very crucial in varieties of ulcers irrespective of the root cause [4]. Therefore, blockers of H^+^, K^+^-ATPase has been considered and explored to design anti-ulcer drugs such as omeprazole, lansoprazole, etc. However, these proton pump blockers are documented to cause lots of side effects [5], especially in the presence of non-steroidal anti-inflammatory drugs, pregnancy, lactation and alcoholic consumption. Current article therefore addresses an alternative source for the potential ulcer cure, addressing the use of common dietary sources for effective prevention or healing of ulcerations (Scheme 1). Further, it is pertinent to address this question because in traditional medicine for which ginger had a high grade; its potency needs to be evaluated *in vivo* in the form it is used in traditional medicine (aqueous extract of ginger—GRAE).

Ginger (*Zingiber officinale* Roscoe.) is cultivated mainly for its rhizome, which is a popular spice in Indian continental cuisine and an equally popular compound in national medicine. The proximate chemical composition of ginger has been shown to contain *∼*1–4% of volatile oils, which are the medically active constituents of ginger. Ginger has been reported to exert anti-oxidant and anti-ulcer [6], anti-inflammatory, anti-tumor [7], carminative, diaphrodic and digestive, expectorant, as well as gastro protective [8] activities. The phenols detected in solvent extracts of ginger were mainly gingerol and zingerone. Recently, we reported that phenolic acids play a major role in inhibiting parietal cell H^+^, K^+^-ATPase, inhibition of an ulcerogen—*H. pylori*, exhibiting anti-oxidative properties *in vitro* [9]. Current data provides evidence for the potential ulcer-preventive ability of phenolics in ginger aqueous extract and addresses the probable mode of action.

## 2. Materials and Methods

### 2.1. Chemicals

Adenosine triphosphate (ATP), glutathione reductase, nitroblue tetrazolium (NBT), 2-thiobarbituric acid (TBA), lanzoprazole were purchased from Sigma Chemical Co. (St Louis, MO, USA). Hexane, hydrochloric acid, trichloroacetic acid (TCA) and solvents used were of the analytical grade purchased from local chemical company (Sisco Research Laboratories, Mumbai, India).

### 2.2. Plant Material and Preparation of Aqueous Extract

Ginger (*Z. officinale* Roscoe.) rhizome was purchased from the local market at Mysore, India and used for studies. One kilogram fresh ginger rhizome was cleaned, washed under running tap water, cut into small pieces, air dried, powdered for particle size of 20 mesh and Ginger powder (10 g) was defatted using hexane in a soxhlet apparatus. One gram of defatted powder was taken in 10 mL distilled water and boiled for 5 min, cooled and centrifuged at 1000 g for10 min. The clear supernatant was separated and referred as ginger aqueous extract (GRAE). A total yield of 8 g/100 g accounting to an average of 8% (w/w) was obtained with triplicate extractions. Obtained aqueous extract was analyzed for bioactivity—anti-oxidants, inhibition of H^+^, K^+^-ATPase/*H. pylori.*

### 2.3. Assessment of Anti-Ulcer Potential of GRAE against Swim/Ethanol Stress-Induced Ulcers

Wistar albino rats weighing about 180–220 g maintained under standard conditions of temperature, humidity and light were provided with standard rodent pellet diet (Amruth feeds, Bangalore, India) and water *ad libitum*. The study was approved by the institutional ethical committee, which follows the guidelines of CPCSEA (Committee for the Purpose of Control and Supervision of Experiments on Animals, Reg. No. 49, 1999), Government of India, New Delhi, India.

All the animals were categorized into two sets of five groups of six numbers each (*n* = 6). GRAE with two doses of 100 and 200 mg kg^−1^ b.w. and lansoprazole 30 mg kg^−1^ b.w. were administered orally twice daily for 14 days. At the end of 14th day animals were fasted for 18 h before inducing ulcer. In the first set ulcer was induced by forced swim stress as per the known protocol [10], while in second set, animals were subjected to ethanol stress [11]. Animals were sacrificed under deep ether anesthesia; stomach/liver was removed and used for enzyme assays. Serum was collected from the blood of all animals and analyzed for various parameters. Ulcer index was determined as described in our previous paper [12]. Stomach and liver tissues were homogenized in chilled Tris-buffer (10 mM, pH 7.4) at a concentration of 5% (w/v). The homogenates were estimated for protein [13], anti-oxidant, anti-oxidant enzymes—catalase, superoxide dismutase (SOD), glutathione peroxidase and TBARS as described previously [14] and compared between groups of animals.

### 2.4. Assessment of H^+^, K^+^-ATPase

Equal weight of gastric tissue from animals of each group was homogenized using Tris-HCl buffer pH 7.4. The gastric membrane vesicles enriched in H^+^, K^+^-ATPase were prepared and the H^+^, K^+^-ATPase activity was assessed as described previously [12].

The enzyme extract (350 *μ*g mL^−1^) was taken in a reaction mixture containing 16 mM Tris buffer (pH 6.5) and the reaction was initiated by adding substrate 2 mM ATP, in addition to 2 mM MgCl_2_ and 10 mM KCl. After 30 min of incubation at 37°C, the reaction was stopped by the addition of assay mixture containing 4.5% Ammonium molybdate and 60% Perchloric acid. Inorganic phosphate formed was measured spectrophotometrically at 400 nm. Enzyme activity was calculated as *μ*moles of inorganic phosphate (Pi) released/h.

### 2.5. Determination of Gastric Mucin

Gastric mucin was isolated from the glandular segments of stomach and quantitated employing a monoclonal anti-human gastric mucin antibody (MAb-GM) by ELISA [15] as well as by Alcian blue dye binding methods [16].

### 2.6. Toxicity Studies

Toxicity studies were carried out in Albino Wistar rats, kept at controlled environment and acclimatized to laboratory conditions for 1 week before study. Rats (180–220 g) were orally fed once daily with GRAE (2 g kg^−1^ b.w.) for 14 days. The control group received the vehicle (distilled water) only. Twenty-four hours after the last dose, number of animals survived were noted and sacrificed by cervical dislocation, blood was collected and serum was used for estimation of TBARS, total protein and enzymes related to liver function tests—serum glutamate pyruvate transaminase (SGPT), serum glutamate oxaloacetate transaminase (SGOT), and alkaline phosphatase (ALP)] using standard protocols [15].

### 2.7. Anti-*Helicobacter pylori* Activity

*Helicobacter pylori* was obtained by endoscopic samples of ulcer patients from KCDC (Karnataka Cardio Diagnostic Centre, Mysore, India) and cultured on Ham's F-12 nutrient agar medium with 5% FBS at 37°C for 2-3 days in a microaerophelic condition. *Helicobacter pylori* culture was characterized by specific tests such as urease, catalase, oxidase, gram staining, colony characteristics and morphological appearance under scanning electron microscope and also confirmed by growth of culture in presence of susceptible and resistant antibiotics.

### 2.8. Agar Diffusion Assay

*Helicobacter pylori* activity was tested by the standard agar diffusion method [17] Briefly, the petriplates were prepared with Ham's F-12 nutrient agar media containing 5% FBS inoculated with 100 *μ*L of *H. pylori* culture (10^5^ cells mL^−1^). Sterile discs of high-grade cellulose of diameter 5.5 mm were impregnated with 20 *μ*L of known extract (0.25–1.0 mg disc^−1^) of GRAE placed on the inoculated petriplates. Amoxicillin was used as positive reference standard and 0.9% saline as negative control. For comparative evaluation discs containing 10 *μ*g each of amoxicillin, GRAE was performed in addition to the control. *Helicobacter pylori* growth inhibition was determined as the diameter of the inhibition zones around the discs. The growth inhibition diameter was an average of four measurements taken at four different directions and all tests were performed in triplicates.

### 2.9. Minimal Inhibitory Concentration

Minimal inhibitory concentration (MIC) values were determined by conventional broth dilution method [17]. Serial dilutions (final volume of 1 ml) of GRAE (50–500 *μ*g mL^−1^) were performed with 0.9% saline. Following this, 9 mL of Ham's F-12 nutrient medium with 5% FBS was added. Broths were inoculated with 100 *μ*L of *H. pylori* suspension (5 × 10^4^ CFU) and incubated for 24 h at 37°C. Amoxicillin was used as a positive control since *H. pylori* is susceptible to amoxicillin and 0.9% saline as negative control. After 24 h, *H. pylori* growth was assayed by measuring absorbance at 625 nm. MIC was defined as the lowest concentration in *μ*g of GAE to restrict the growth to <0.05 absorbance at 625 nm (no macroscopic visible growth).

### 2.10. Scanning Electron Microscopy

The bacterium was grown overnight in broth at 37°C and 100 *μ*L (8 log_10_ CFU mL^−1^) in 5 mL broth medium were incubated with amoxicillin (10–30 *μ*g mL^−1^) or GRAE (50–200 *μ*g mL^−1^) and major phenolic acids such as cinnamic, gentisic, ferulic and gallic acids (10–50 *μ*g mL^−1^) for 6 h at 37°C and the suspension without treatment was taken as control. After incubation, 100 *μ*L aliquot was processed for scanning electron microscopic studies as described earlier [18]. Multiple fields of visions were viewed and results were documented by photography at different magnifications.

### 2.11. HSA-Phenolics Interaction Studies

Stock solution of human serum albumin was prepared to a concentration of 1.0 × 10^−4^ M in Tris-HCl buffer of pH 7.4 containing 100 mM sodium chloride. All the phenolic compounds were prepared to a concentration of 10 mg/100 mL in ethanol (95%) because ethanol has no fluorescence and does not affect the determinations. All fluorescence measurements were made in a Shimadzu RF-5301PC spectrofluorophotometer.

A series of assay solutions were prepared by adding 10 *μ*L of the stock solution of HSA and varied concentrations of phenolics (0.5–2.5 *μ*g mL^−1^) into each marked tube respectively, and diluted to the mark 1.0 mL with Tris-HCl buffer of pH 7.4. The concentration of HSA was constant and the possible interaction was studied at different concentrations of phenolic acids. Tubes were mixed thoroughly and placed in the thermostat water-bath at 37°C for 5 min, and transferred to the quartz cuvette and fluorescence emission spectra were recorded in the wavelength range 290–500 nm by exciting HSA at 280 nm using a slit width of 5/5 nm. Wavelength nearer to shift observed was recorded to understand the involvement of tryptophan/tyrosine residue in HSA and were expressed as Sterner's constant.

### 2.12. Measurement of Anti-Oxidant Activity in GRAE

#### 2.12.1. Free Radical Scavenging Activity

The anti-oxidant activity of GRAE on the basis of the scavenging activity of the stable 1,1-diphenyl-2-picrylhydrazyl (DPPH) free radical, was determined by the method described by Braca et al. [19]. An aliquot of 100 *μ*L of GRAE at various concentrations- 2.5–15 *μ*g mL^−1^ were added to 3 mL of 0.004% methanol solution of DPPH. The mixture was shaken vigorously and left to stand for 20 min at room temperature in the dark. The absorbance of the resulting solution was measured spectrophotometrically at 517 nm. The capability to scavenge the DPPH radical was calculated using the following equation.

(1) $$\;\text{Scavenging}\;\;\text{ effect (}\%)=\frac{\text{Absorbance of control at 517 nm}-\text{Absorbance of sample at 517 nm }}{\text{Absorbance of control at 517 nm}}\times 100,$$

#### 2.12.2. Reducing Power Ability

The reducing powers of GRAE were determined according to the method of Yen and Chen [20]. The extract of GRAE (5–25 *μ*g mL^−1^) were mixed with an equal volume of 0.2 M phosphate buffer, pH 6.6 and 1% potassium ferricyanide. The mixture was incubated at 50°C for 20 min. An equal volume of 10% TCA was added to the mixture, centrifuged at 3000 g for 10 min. The upper layer of solution was mixed with distilled water and 0.1% FeCl_3_ at a ratio of 1 : 1 : 2 (v/v/v) and the absorbance was measured at 700 nm. Increased absorbance of the reaction mixture indicated increased reducing power.

#### 2.12.3. Inhibition of Lipid Peroxidation of Rat Liver Homogenate

*In vitro* lipid peroxidation levels in rat liver homogenate was measured as TBARS. Ten percent of fresh liver homogenate was prepared in 20 mM phosphate buffer saline (PBS), pH 7.4 (36). Briefly, 0.25 mL of liver homogenate was incubated with 5–25 *μ*g mL^−1^ of GRAE in 20 mM PBS, pH 7.4. After 5 min of pre-treatment, 0.5 mL each of ferric chloride (400 mM) and ascorbic acid (400 mM) was added and incubated at 37°C for 1 h. The reaction was terminated by addition of 2.0 mL of TBA reagent (15% TCA, 0.37% TBA in 0.25 N HCl) and tubes were boiled for 15 min at 95°C, cooled, centrifuged and read at 532 nm. TBARS was measured by using a standard TMP (1,1,3,3 tetramethoxy propane) calibration curve (0.1–0.5 *μ*g) and expressed as percent inhibition of lipid peroxidation by extracts.

#### 2.12.4. DNA Protection Assay

The DNA-protective effect of phenolic fractions was determined electrophoretically (Submarine electrophoresis system, Bangalore Genei, Bangalore, India) using calf thymus DNA (37). Calf thymus DNA (1 *μ*g in 15 *μ*L) was subjected to oxidation by Fenton's reagent (30 mM H_2_O_2_, 50 mM ascorbic acid and 80 mM FeCl_3_). Relative difference in the migration between the native and oxidized DNA was ensured on 1% agarose gel electrophoresis after staining with ethidium bromide. Gels were documented (Herolab, Germany) and the intensity of the bands was determined (Easywin software). Protection to DNA was calculated based on the DNA band corresponding to that of native in the presence and absence of 2 and 4 *μ*g of GRAE.

### 2.13. Determination of the Phenolic Content and Composition in GRAE

The total phenolic content in GRAE was determined using Folin-Ciocalteu reagent as described earlier [21]. Gallic acid was used as standard for the generation of calibration curve. Total phenolic content was expressed as Gallic Acid Equivalents (GAE) in mg g^−1^ of GRAE.

Phenolic acids from GRAE were analyzed by HPLC (model LC-10A. Shimadzu Corp, Kyoto, Japan) on a reversed phase Shimpak C_18_ column (4.6 × 250 mm, Shimadzu Corp, Kyoto, Japan) using a diode array UV-detector (operating at *λ*_max_ 280 nm). A solvent system consisting of water/acetic acid/methanol (isocratic, 80 : 5 : 15 v/v/v) was used as mobile phase at a flow rate of 1 mL min^−1^ [22]. Phenolic acid standards such as caffeic, coumaric, cinnamic, ferulic, gallic, gentisic, protocatechuic, syringic and vanillic acids were employed for identification of phenolic acids present in GRAE by comparing the retention time under similar experimental conditions.

### 2.14. Statistical Analysis of Data

All the experiments were carried out in triplicates and the results are expressed as mean value ± SD. *P*-value was calculated by the Mann-Whitney test. Duncan's New Multiple Range Test was performed to understand the degree of significance between controls and treated samples.

## 3. Results

### 3.1. Ulcer-Preventive Effect of GRAE in Swim-Stress-/Ethanol-Induced Ulcer Animal Model

**Figure 1:**
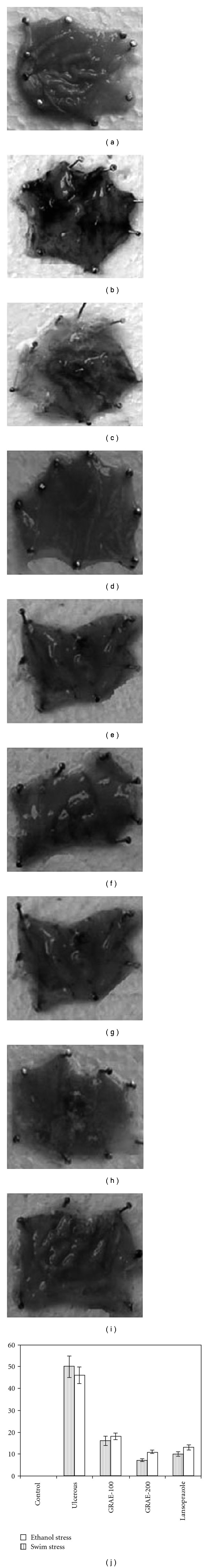
Macroscopic observation of Ulcers in ulcer induced/protected stomachs in swim stress-/ethanol stress-induced ulcer models. Ulcer was induced in animals by either swim stress (SS) or ethanol stress (ES) in group of pre-treated/untreated animals at indicated concentrations. In healthy control (a) no ulcer lesions or damage in the stomach tissue was observed. In ethanol stress (b) and swim stress (c) induced animals, ulcers score were very high. Lansoprazole (d, g) and GRAE at 100 and 200 mg kg^−1^ treated animals showed dose-dependent reduction in stomach lesions (e, f, h, i). (j) Maximum ulcer index observed during stress induction was controlled in a concentration-dependent manner. Reduction in ulcer index and percent protection is depicted.

Ulcer-preventive effect of GRAE was evaluated by using swim/ethanol stress-induced ulcers. These two models are well-accepted oxidative stress-induced ulcerations model. Mechanism of induction although in both the cases mediated by reactive oxygen species (ROS), in swim stress, it is more through initiation of parietal cell- H^+^, K^+^-ATPase activation, while in ethanol it is via damage of mucosal epithelium.  Figure 1(a) depicts the stomach of healthy rat which showed no damage or lesions. In swim/ethanol stress-induced ulcers, the lesions were characterized by multiple hemorrhagic red bands of different size along the long axis of the glandular stomach (Figures 1(b) and 1(c)). Oral treatment of GRAE at 100 and 200 mg kg^−1^ b.w. as well as lansoprazole at 30 mg kg^−1^ b.w. showed protection in a dose-dependent manner with no intraluminal bleeding and insignificant number of gastric lesions (Figures 1(e), 1(f), 1(h) and 1(i)). Quantitative reduction in ulcer index in treated rats compared to either ulcer induced or healthy is calculated and depicted in Figure 1(j). Data indicated that GRAE protected dose dependently up to 77–86% protection at 200 mg kg^−1^ b.w.

### 3.2. Evaluation of GRAE Potential on Oxidant and Anti-Oxidant Status in Ulcerous and Treated Animals

A 2- to 2.4-fold increase in SOD and GPX levels in stomach tissue were observed in swim/ethanol stress-induced animals and were normalized upon treatment with GRAE in a dose-dependent manner. Whereas, CAT and GSH decreased to 1.6-fold during stress-induced ulcerous conditions were normalized upon treatment with GRAE as shown in Tables 1 and 2. Approximately 2.6-fold increase in TBARS levels indicated the lipid peroxidation or damage of stomach tissue in ulcerous animals; and was recovered up to 91% upon treatment with GRAE. A 2- to 2.3-fold increase in TBARS levels observed in serum and liver homogenate of stress-induced ulcerous groups was recovered also up to 75% upon GRAE treatment at 200 mg kg^−1^ b.w.

### 3.3. H^+^, K^+^-ATPase Inhibition and Mucin Protection by GRAE

The elevated levels of H^+^, K^+^-ATPase in swim stress and ethanol stress was normalized upon treatment with GRAE in a dose-dependent manner. Oral pre-treatment of GRAE inhibited the H^+^, K^+^-ATPase activity and showed 3.1- and 2.9-fold reduction at 200 mg kg^−1^ b.w. In case of lansoprazole, slightly decreased level of H^+^, K^+^-ATPase activity was observed (Table 3) and the results were also validated by *in vitro* assay—inhibition of H^+^, K^+^-ATPase enzyme from sheep stomach parietal cells. GRAE inhibited H^+^, K^+^-ATPase activity with an IC_50_ of 16.5 ± 1.2 *μ*g GAE as opposed to that of lansoprazole (19.3 ± 2.2 *μ*g w/w) indicating increased potency of GRAE. Further, the damaged mucin in ulcerous condition was protected up to 68–72% upon treatment with GRAE at 200 mg kg^−1^ b.w (Table 3).

### 3.4. Toxicity Studies with GRAE

Toxicity studies with aqueous solution of GRAE were carried out in rats for safety evaluation indicated no lethal effect upto 1  g kg^−1^ b.w. when orally fed for 14 days. There were no significant differences in total protein, TBARS levels, SGPT, SGOT and ALP between normal and GRAE-treated rats (Table 4) indicating no adverse effect on the major organs. Animals after above treatment schedule remained healthy as that of control animals with normal food and water intake, body weight gain and behavior.

### 3.5. Anti-Helicobacter pylori Activity of GRAE

The bacteria isolated from endoscopic samples were Gram-negative, motile and showed positive for urease, catalase and oxidase tests (Table 5). Further, it was confirmed by the response to antibiotics as it was resistant to antibiotics like erythromycin, nalidixic acid, polymixin B, penicillin and vancomycin and was susceptible to amoxicillin, clarithriomycin and metronidazole. The appearance of a characteristic white mucilaginous colony confirms the identity of bacteria as *H. pylori*.

**Figure 2:**
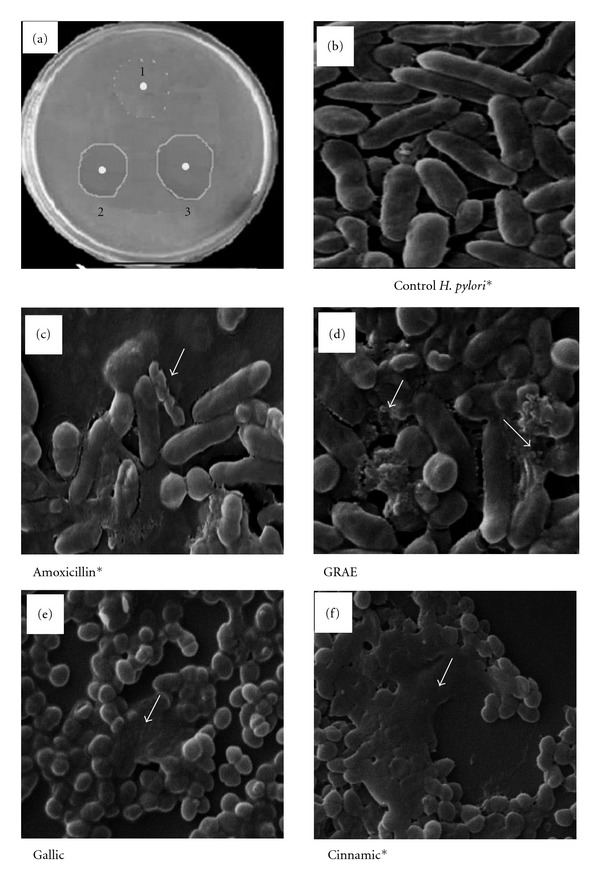
Effect of GRAE on *H. pylori* growth. Anti-*H. pylori* activity was tested by the standard agar diffusion method (a). A 5.5 mm discs containing 10 *μ*g each of Amoxicillin-a known antibiotic (a.2); GRAE were impregnated with agar and (a.1) served as control with no inhibitor disc. Clear area around the disc represents the inhibition zone due to the effect of the test fraction. (b)–(f) indicate the scanning electron microscopic pictures at 15 k magnification of control (b), amoxicillin (c), GRAE (d) treated *H. pylori* and (e) and (f) depicts the *H. pylori* treated with pure phenolic acids gallic and cinnamic acid respectively. Untreated control cultures indicate uniform rod shaped *H. pylori* cells. Amoxicillin treatment showed coccoid form, blebbing, fragmented and lysed cells. Similar altered conditions observed in GRAE and pure phenolics treated *H. pylori* cells. *Figures were taken from our previous paper [14] for the comparative purpose.

In the agar diffusion method, GRAE showed a clear inhibition zone around the disc at 50 *μ*g mL^−1^ concentration equivalent to that of a susceptible antibiotic amoxicillin at 10 *μ*g mL^−1^ (Figure 2(a)). MIC values determined by broth dilution method indicated significant anti-*H. pylori* activity at 300 ± 38 *μ*g mL^−1^ at *P* ~ .003.

SEM observation revealed the efficacy of GRAE action in inhibiting the *H. pylori g*rowth. Figure 2(b) shows the uniform rod-shaped normal *H. pylori* cells, whereas the cells treated with amoxicillin, GRAE, gallic and cinnamic acid changed from helical form to coccoid and became necrotic (showed with arrows in Figures 2(c)–2(f)). Coccoid form with blebs in the bacterial surface, appearance of vacuoles, granules and an area of low electron density in the cytoplasm (showed with arrow marks) were observed in GRAE-treated sample indicating the lysis of *H. pylori.* Results were also substantiated by viability test, which indicated the loss of 85% viability upon treatment with GRAE supporting anti-microbial nature of GRAE.

### 3.6. Multipotent Anti-Oxidant Activity of GRAE

**Figure 3:**
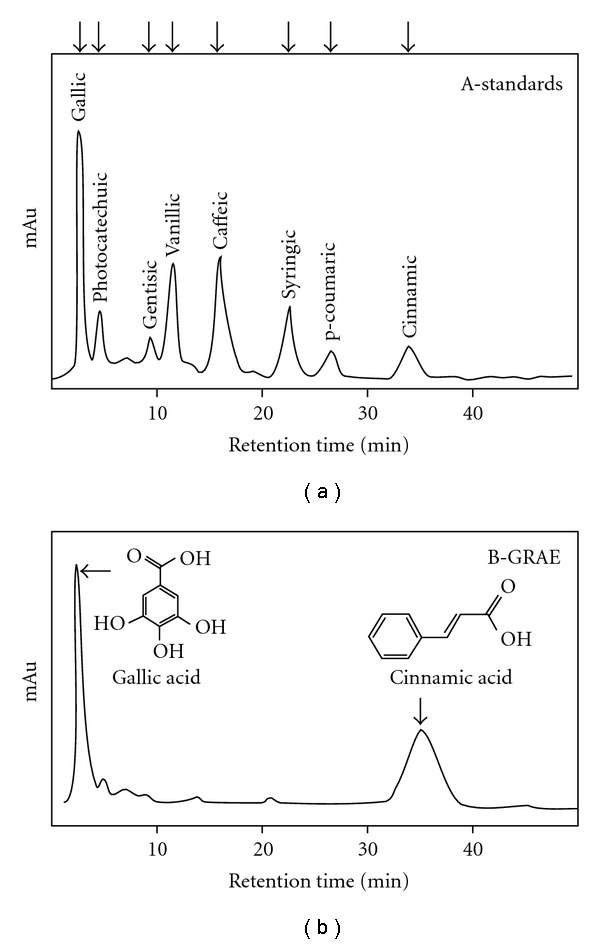
HPLC analysis of phenolic acid constituents in GRAE. A 1 mg mL^−1^ solution of GRAE (a.3) was prepared, after vortexing for 10 min at room temperature with the mobile phase-water/acetic acid/methanol—80 : 5 : 15 (v/v/v)—Isocratic and 20 *μ*L of each was loaded on to HPLC Shimpak C18 column (4.6 × 250 mm, Shimadzu Corp, Kyoto, Japan). A 20 *μ*L of mg mL^−1^ standard phenolic acids were loaded independently and their specific retention time (min) was established. Phenolic acids in GRAE were identified comparing with their retention time with known standards.

**Figure 4:**
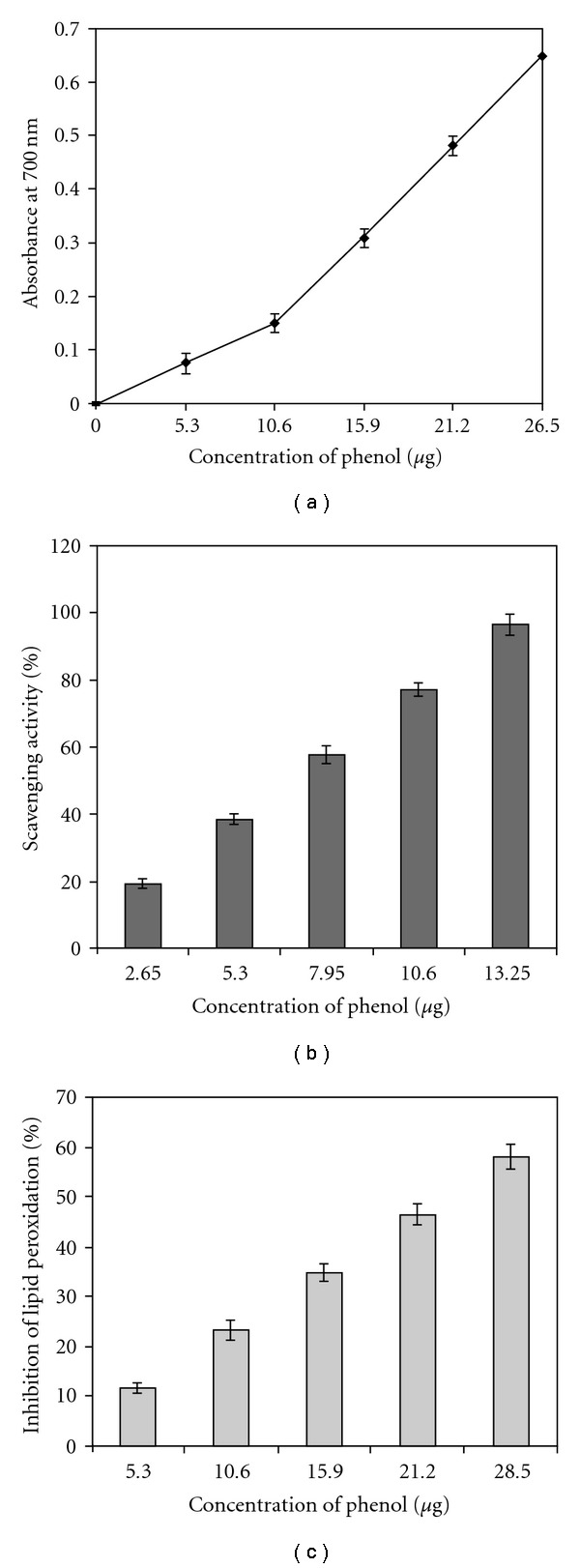
Anti-oxidant potency of GRAE. Concentration of 5–25 *μ*g GAE/mL of GRAE were examined for reducing power (a), free radical scavenging (b) and inhibition of lipid peroxidation (c) as per the protocol described under materials and methods section. All data are the mean ± SD of three replicates.

**Figure 5:**
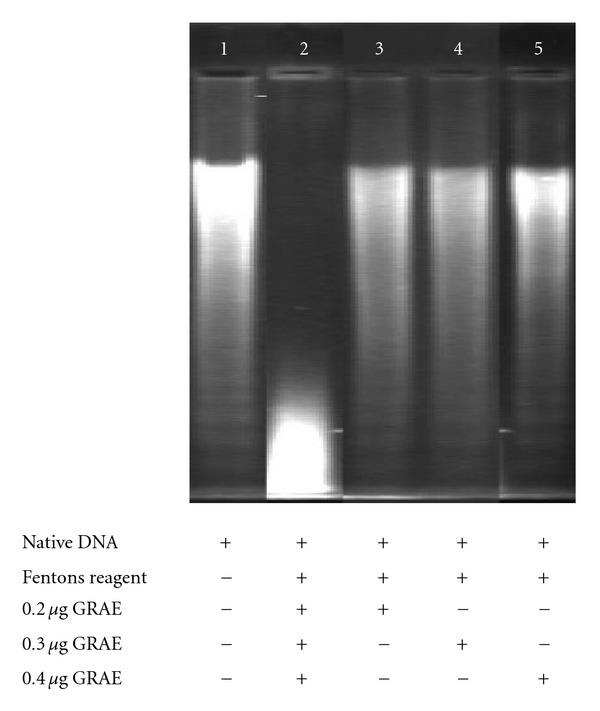
DNA protection ability of GRAE. One microgram of native calf thymus DNA in (lane 1); DNA treated with Fenton's reagent (lane 2); DNA pre-treated with 2, 4, 6 *μ*g of GRAE (lanes 3–5) were loaded on to the 1% agarose gel. Bands were visualized by staining with ethidium bromide and in the transilluminator increased mobility represents DNA damage.

The ginger aqueous extract possessed 7.6 ± 0.5 mg GAE/g phenolics. The HPLC analysis of GRAE revealed that cinnamic acid (50%) and gallic acid (46%) were the major phenolic acids with small amounts of caffeic, ferulic, gentisic, protocatechuic, syringic and vanillic acids (Figure 3). The total reducing power ability of GRAE was ∼1168 ± 90 U g^−1^ GAE (Figure 4(a)) and Figures 4(b) and 4(c) illustrates the scavenging effect of GRAE on DPPH radical and inhibition of lipid peroxidation at IC_50_ 6.8 ± 0.4 *μ*g mL^−1^ GAE and 16.8 ± 1.2 *μ*g GAE, respectively. GRAE also exhibited DNA protective ability (Figure 5); the damaged DNA migrated fast, while protected DNA moved slowly as that of normal, untreated DNA. Image analysis indicated recovery of DNA up to >90%.

### 3.7. HSA Interaction

Since there was a significant reduction in H^+^, K^+^-ATPase which could be attributed to phenolics^6^, current study attempted to explore the possible binding of phenolics to the enzyme by virtue of phenolic acids. For comparative purpose, two phenolic acids—gallic acid and cinnamic acids that showed poorer and potent H^+^, K^+^-ATPase inhibitory activity respectively, were examined in presence and absence of gallic/cinnamic acids and expressed as Sterner's constant.

**Figure 6:**
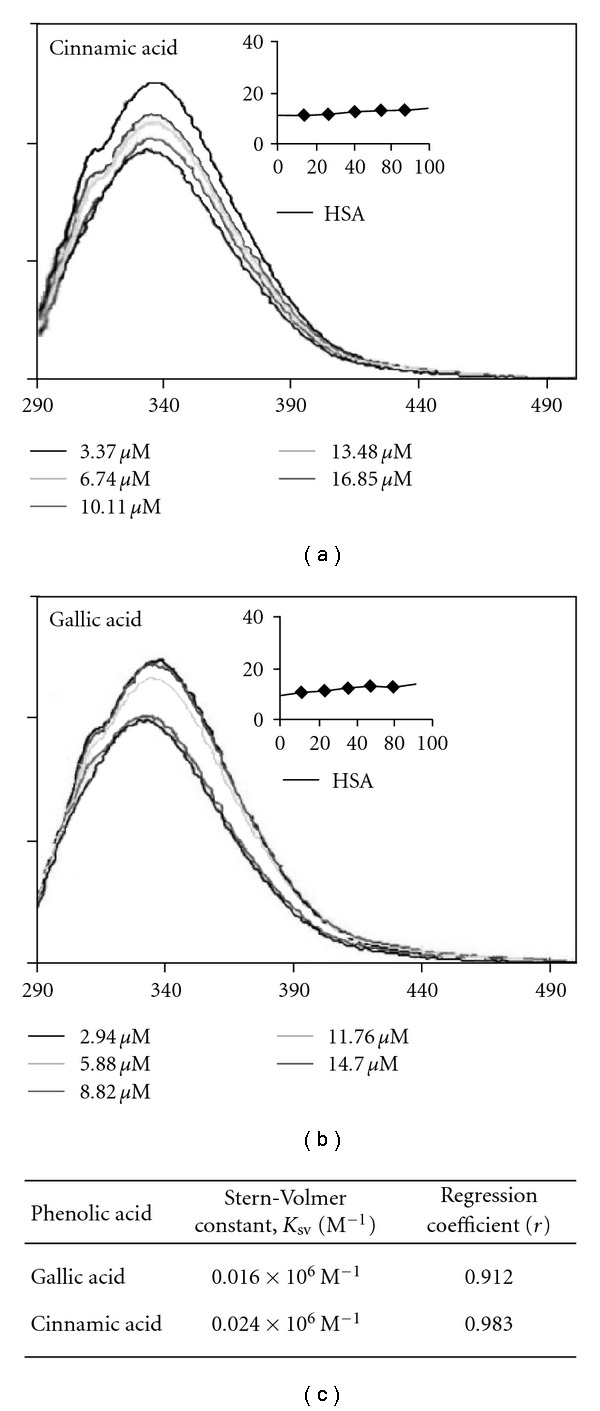
Fluorescence emission spectra of HSA in the presence of gallic acid and cinnamic acid. The extinction wave length was 280 nm. Both excitation and emission slits widths were 5 nm. Inlaid figure—Stern-Volmer plots (*x*-axis: concentration in micrograms; *y*-axis: *F* ^0^/*F*).

Results from HSA interaction studies indicated that the changes occurred in the environment of tryptophan residues in HSA and was dependent on the applied phenolic acids. As shown in Figure 6, both gallic and cinnamic acids showed HSA binding, but to varying extent. *K*_sv_ of cinnamic and gallic acid were found to be 0.024 × 10^6^ M^−1^ and 0.016 × 10^6^ M^−1^, respectively. Approximately 1.5-fold increase in binding was observed with cinnamic acid than gallic acid. However, 5-fold better H^+^, K^+^-ATPase inhibition with cinnamic acid than gallic acid suggests that parameters other than binding may also influence H^+^, K^+^-ATPase inhibitory activity in case of cinnamic acid. Cinnamic acid being hydrophobic, may access the membrane domain of H^+^, K^+^-ATPase which is lacking in HSA, may possibly accounted for enhanced inhibitory activity.

## 4. Discussion

Ulcer results from an imbalance between aggressive factors and the maintenance of mucosal integrity through the endogenous defense mechanisms. To regain the balance, different therapeutics including spice and plant extracts have been used. In the previous paper, we had shown that free and bound phenolics of ginger possessed potential ulcer preventive activity *in vitro*, including inhibition of H^+^, K^+^-ATPase and *H. pylori* growth [9]. However, in view of addressing a question whether the traditional practice of using crude ginger extract in either boiled water or cold water extract can yield compounds which are gastroprotective in nature; we evaluated *in vitro* and *in vivo* ulcer-preventive properties of GRAE and determined whether it also contained phenolic acids that favors gastroprotection as reported in our previous papers [9, 17].

ROS are implicated in the pathogenesis of several diseases. Free radicals are continuously produced during normal physiologic events and removed by anti-oxidant defense mechanisms, including enzymes such as SOD, CAT and enzymes involved in the glutathione redox cycle. Free radicals cause lipid peroxidation and production of highly toxic lipid derivatives, which in turn can modify cell functions and even may lead to cell death. Oxidative modification of proteins may result in structural impairment and also change their functional properties such as their involvement in signaling, critical for numerous cellular functions. They affect the vasomotor function of vasculature throughout the body via alterations in the activity of the autonomic nervous system, thus changing the blood flow to involve tissues such as mucosa. Oxidative stress (OS) being major source in causing ROS-mediated ulceration, up/down regulation of anti-oxidant/anti-oxidative enzymes reveal the ability of GRAE to counteract the OS condition and hence protection to ulcer.

GRAE at 200 mg kg^−1^ b.w. protected swim stress/ethanol-induced ulcer lesions up to 86% similar to that of lansoprazole (80%), a known antiulcer drug at 30 mg kg^−1^ b.w. Bloody streaks, inflammations, oozing of blood into the lumen of the stomach, and so forth, observed in ulcerous animals were not found in GRAE ingested animals, similar to those of healthy rats indicating the gastroprotective effect of GRAE. Further, we followed the protective effect investigating the biochemical parameters such as alterations in the gastric mucin, oxidants, GSH, H^+^, K^+^-ATPase and anti-oxidant enzymes level including CAT, SOD, peroxidase, and so forth, in the ulcerated organ—stomach as well in the metabolizing organ—liver in all groups of rats—healthy, ulcerated and GRAE/lansoprazole treated. Preventive anti-oxidant enzymes such as SOD and CAT are the first line of defense against ROS. Administration of GRAE resulted in a significant increase in the SOD, CAT and reduced GSH levels (Tables 1 and 2) similar to those of control animals, suggesting the efficacy of GRAE in preventing free radical-induced damage during ulceration. SOD, GPx, CAT and GSH all contribute to maintain anti-oxidant status during oxidative stress condition such as ulcers. SOD and GPx levels were increased during ulceration to scavenge superoxide and peroxy radicals generated during ulceration. Depletion in CAT and reduced GSH suggest the utilization of these components towards the neutralization of increased free radicals.

In our experimental model, ∼3.4-fold reduction in gastric mucin and 2.4-fold reduced glutathione as well as 2.6-fold increased oxidative product—TBARS in the stomach were normalized by GRAE (Tables 1–3) treatment. Gastric ulcers are often a chronic disease and it may persist for 10–20 years as characterized by repeated episodes of healing and re-exacerbations [23]. Stress-induced ulcer better resembles clinical ulcers in chronicity, severity and practicality of experiencing stress due to varietal patterns of lifestyle in day to day life and serves the most reliable model to study ulcer healing process [24, 25]. The incidence of swim stress-induced ulcer is predominant in the glandular part of the stomach leading to gastric mucosal/mucin damage. GRAE significantly prevented ulcers both by reducing the oxidative stress as well as boosting the mucosal defense.

Further, during our study, we evaluated the possible mechanism of protection to gastric ulcer apart from up-regulation of anti-oxidant and anti-oxidant enzyme levels. Gastric H^+^, K^+^-ATPase located in the apical membrane of parietal cells, pumps protons into the gastric lumen, using energy derived from the hydrolysis of ATP, and is thus involved in gastric acid secretion. Accordingly, the activity of gastric H^+^, K^+^-ATPase was measured in the stomach homogenate, which showed 3-fold up-regulation of the enzyme in ulcer condition and was normalized by treatment with GRAE (Table 3). Results were further substantiated by sheep H^+^, K^+^-ATPase inhibition by GRAE with an IC_50_ of 16.5 ± 1.2 *μ*g mL^−1^ on par or better than lansoprazole (19.3 ± 2.2 *μ*g w/w), indicating the potential multi-targeted effect of GRAE in preventing swim stress-induced ulcers in experimental rats. GRAE may find itself more useful as a H^+^, K^+^-ATPase (proton pump) inhibitor than the existing pump inhibitors, since they have adverse effects as reported particularly under conditions of pregnancy/lactation and alcohol or any other drug consumption. Least toxicity of GRAE may also find GRAE as useful alternative source for ulcer healing therapeutics.

Besides, GRAE also exhibited the anti-*H. pylori* activity. The MIC values obtained confirmed the significant (*P* = .003) anti-*H. pylori* activity, and as reported in previous papers results were supported by an observation of Vattem et al. [18], where phenolic phytochemicals such as cinnamic acid, cinnamaldehyde, coumarins and flavonoids have been suggested to exhibit high anti-*H. pylori* activity. GRAE with higher content of cinnamic acid showed better inhibition of *H. pylori*. As mentioned in earlier studies, phenolics were thought to exert their anti-microbial effect by causing (i) hyperacidification at the plasma membrane interface of the microorganism [26] or (ii) intracellular acidification, resulting in disruption of H^+^, K^+^-ATPase required for ATP synthesis of microbes [27] or (iii) may be related to inactivation of cellular enzymes causing membrane permeability changes [27].

Also, it is intriguing to observe that cinnamic acid is acting as a potent inhibitor of H^+^, K^+^-ATPase, and also *H. pylori* probably by stronger binding of cinnamic acid to membrane domains of H^+^, K^+^-ATPase and *H. pylori* than gallic acid, which is a poor inhibitor of H^+^, K^+^-ATPase and *H. pylori* (Figure 6). Lack of correlation between fold inhibitory activity versus binding between gallic/cinnamic acid still do not rule out the proposed mechanism. Because being hydrophobic cinnamic acid binding ability may be higher with H^+^, K^+^-ATPase and *H. pylori* which carry membrane domains than HSA alone which is devoid of this hydrophobic domain.

GRAE also exhibited reducing power and prevented free radical-induced lipid and DNA peroxidation. This anti-oxidative property also contributes significantly to reduce ulcer condition and justifies the ethno medical claims.  Table 6 shows the relative concentration of individual phenolic acids towards the antiulcer activity. Our data in the present and previous papers indicate that cinnamic acid, caffeic acid and p-coumaric acids contribute significantly to inhibition of H^+^, K^+^-ATPase and *H. pylori* growth in the free as well as in the bound form. Recently, we also reported the role of cinnamic acid in cross-linking property of pectic polysaccharide and its contribution hence to the structural stability (manuscript communicated).

## 5. Conclusions

The present study clearly demonstrated that aqueous extract of ginger was able to protect the gastric mucosa from stress-induced mucosal lesions and inhibits gastric acid secretion probably by blocking H^+^, K^+^-ATPase action, inhibiting growth of *H. pylori* and offering anti-oxidant protection against oxidative stress-induced gastric damage The results confirm the popular use of ginger for its medicinal properties in Ayurveda and folklore medicines. These results further suggest the use of ginger for gastric disorders that needs to be considered as possibilities for new therapeutic approaches.

## Figures and Tables

**Table 1:** Anti-oxidant/anti-oxidant enzymes and TBARS levels in swim stress-induced ulcer model (*n* = 6) mean ± SD.

Parameters	Protein (mg g^−1^)	SOD (U mg^−1^)	Catalase (U mg^−1^)	Glutathione Peroxidase (nmol g^−1^)	GSH (U mg^−1^)	TBARS nmol
Stomach						
Healthy	2.23^c^ ± 0.16	092.9^a^ ± 08	46.5^c^ ± 4.6	28.6^a^ ± 2.4	376.6^c^ ± 37	0.82^a^ ± 0.07
Ulcerated	1.39^a^ ± 0.16	201.3^d^ ± 21	22.8^a^±2.1	68.6^c^ ± 5.6	216.2^a^ ± 23	2.16^c^ ± 0.19
GRAE 100 mg kg^−1^	1.68^a^ ± 0.16	161.6^c^ ± 18	38.6^b^ ± 3.4	32.4^b^ ± 3.5	306.5^b^ ± 32	1.12^b^ ± 0.08
GRAE 200 mg kg^−1^	2.46^b^ ± 0.23	136.4^b,c^ ± 14	43.1^b,c^ ± 4.5	26.9^a^ ± 2.8	351.5^c^ ± 34	0.91^a^ ± 0.06
Lansoprazole	2.13^b^ ± 0.13	124.3^b^ ± 14	44^b,c^ ± 4.5	26.7^a^ ± 2.3	325^b,c^ ± 32	0.94^a^ ± 0.08
Serum						
Healthy	6.621^a^ ± 0.51	112.3^a^ ± 28	44.20^c^ ± 4.9	0.221^a^ ± 0.004	23.6^c^ ± 3.0	0.165^a^ ± 0.01
Ulcerated	6.845^a^ ± 0.53	264.6^d^ ± 32	22.90^a^ ± 3.1	0.286^c^ ± 0.02	11.1^a^ ± 1.8	0.326^d^ ± 0.02
GRAE 100 mg kg^−1^	6.663^a^ ± 0.62	186.8^c^ ± 21	34.23^b^ ± 3.6	0.293^d^ ± 0.03	16.5^b^ ± 1.7	0.264^c^ ± 0.02
GRAE 200 mg kg^−1^	6.943^a^ ± 0.61	148.6^b^ ± 15	41.45^c^ ± 4.3	0.254^b^ ± 0.03	22.3^b,c^ ± 2.3	0.186^a,b^ ± 0.02
Lansoprazole	6.632^a^ ± 0.62	143.6^b,c^ ± 16	36.82^b^ ± 3.4	0.246^a^ ± 0.02	18.8^a^ ± 2.3	0.188^b^ ± 0.01
Liver						
Healthy	24.2^c^ ± 0.31	261.5^b^ ± 41	28.42^d^ ± 3.1	0.32^a^ ± 0.02	414^c^ ± 51	0.98^a^ ± 0.13
Ulcerated	21.9^a^ ± 0.23	142.4^a^ ± 18	22.18^b,c^ ± 2.6	0.58^c^ ± 0.05	221^a^ ± 26	2.41^d^ ± 0.23
GRAE 100 mg kg^−1^	23.7^b^ ± 0.27	196.6^a^ ± 21	22.54^b,c^ ± 2.4	0.45^a,b^ ± 0.04	323^b^ ± 33	1.98^c^ ± 0.21
GRAE 200 mg kg^−1^	24.2^b^ ± 0.23	266.7^d^ ± 36	26.67^a^ ± 2.4	0.46^a^ ± 0.04	382^a^ ± 36	1.45^b^ ± 0.27
Lansoprazole	23.7^b^ ± 0.25	234.4^c,d^ ± 24	24.62^a^ ± 2.3	0.41^a^ ± 0.03	325^a^ ± 31	1.64^b^ ± 0.21

Different letters “a” to “d” in the column represents that values are significantly different when compared between ulcer induced with healthy control and GRAE/lansoprazole-treated groups.

**Table 2:** Anti-oxidant/anti-oxidant enzymes and TBARS levels in swim ethanol-induced ulcer model (*n* = 6) mean ± SD.

Parameters	Protein (mg g^−1^)	SOD (U mg^−1^)	Catalase (U mg^−1^)	Glutathione Peroxidase (nmol g^−1^)	GSH (U mg^−1^)	TBARS nmol
Stomach						
Healthy	2.23^a^ ± 0.21	078.8^a^ ± 07	48.2^c^ ± 6.2	26.5^a^ ± 2.3	368.2^c^ ± 42	0.76^a^ ± 0.06
Ulcerated	2.32^a^ ± 0.09	218.3^d^ ± 20	21.6^a^ ± 2.2	76.6^c^ ± 6.0	208.4^a^ ± 21	1.93^c^ ± 0.21
GRAE 100 mg kg^−1^	2.38^a^ ± 0.24	156.9^c^ ± 16	36.1^b^ ± 3.8	56.9^b^ ± 6.4	286.6^b^ ± 27	0.96^b^ ± 0.08
GRAE 200 mg kg^−1^	2.42^a^ ± 0.26	128.3^b^ ± 11	39.2^b^ ± 4.1	28.3^a^ ± 3.1	342.2^c^ ± 36	0.87^ab^ ± 0.10
Lansoprazole	2.42^a^ ± 0.19	168.6^c^ ± 1.6	38.2^b^ ± 1.4	25.2^a^ ± 2.03	252^b^ ± 16	0.96^ab^ ± 0.2
Serum						
Healthy	3.62^a^ ± 0.51	112.3^a^ ± 28	44.20^c^ ± 4.9^a^	0.221^a^ ± 0.04	23.6^d^ ± 3.0	0.165^a^ ± 0.01
Ulcerated	6.52^a^ ± 0.69	282.3^d^ ± 26	28.36^a^ ± 3.2^b^	0.315^c^ ± 0.03	09.6^a^ ± 1.2	0.465^d^ ± 0.03
GRAE 100 mg kg^−1^	6.58^a^ ± 0.62	198.6^c^ ± 22	33.45^ab^ ± 4.1^b^	0.264^b^ ± 0.02	15.4^c^ ± 1.2	0.312^c^ ± 0.03
GRAE 200 mg kg^−1^	6.62^a^ ± 0.67	136.4^b^ ± 18	42.34^b^ ± 3.3^a^	0.251^b^ ± 0.02	22.5^c^ ± 2.1	0.172^a^ ± 0.02
Lansoprazole	6.32^a^ ± 0.69	210.7^c^ ± 28	34.12^ab^ ± 4.6^b^	0.252^b^ ± 0.03	14.6^b^ ± 1.6	0.214^ab^ ± 0.02
Liver						
Healthy	24.2^a^ ± 0.31	261.5^b^ ± 1.1	28.42^c^ ± 3.1	0.32^b^ ± 0.02	414^c^ ± 51	0.98^a^ ± 0.13
Ulcerated	24.3^a^ ± 0.31	118.1^a^ ± 16	19.64^b^ ± 2.2	0.48^b,c^ ± 0.03	392^b,c^ ± 41	2.98^d^ ± 0.31
GRAE 100 mg kg^−1^	26.4^a^ ± 0.23	127.4^a^ ± 12	22.32^b^ ± 2.3	0.43^b^ ± 0.04	365^b^ ± 34	2.63^c^ ± 0.24
GRAE 200 mg kg^−1^	26.8^a^ ± 0.25	238.3^c^ ± 24	25.23^a^ ± 2.6	0.36^a^ ± 0.03	396^a,b^ ± 36	1.36^b^ ± 0.13
Lansoprazole	26.8^a^ ± 0.29	254.5^b^ ± 26	14.24^a^ ± 1.8	0.31^a^ ± 0.03	211^a^ ± 28	1.61^b^ ± 0.16

Different letters “a” to “d” in the column represents that values are significantly different when compared between ulcer induced with healthy control and GRAE/lansoprazole-treated groups.

**Table 3:** Gastric mucin and H^+^, K^+^-ATPase levels in healthy, ulcerated and protected rats (*n* = 6) mean ± SD.

Group (*n* = 6)	Mucin content (mg g^−1^)	H^+^, K^+^-ATPase *μ*mol Pi released mg^−1^ h^−1^
Healthy	62.05^d^ ± 5.1	0.721^a^ ± 0.02
Swim stress-induced ulcer model		
Swim stress induced	18.42^a^ ± 3.4	2.610^d^ ± 0.21
GRAE 100 mg kg^−1^ b.w.	43.36^b^ ± 3.6	1.316^c^ ± 0.18
GRAE 200 mg kg^−1^ b.w.	48.41^b,c^ ± 3.4	0.831^a^ ± 0.14
Lansoprazole 30 mg kg^−1^ b.w.	35.14^b^ ± 2.4	1.220^b^ ± 0.12
Ethanol stress-induced ulcer model		
Ethanol stress induced	22.37^a^ ± 2.3	2.318^c^ ± 0.24
GRAE 100 mg kg^−1^ b.w.	36.32^b^ ± 3.6	1.213^b^ ± 0.26
GRAE 200 mg kg^−1^ b.w.	46.54^c^ ± 3.8	0.793^a^ ± 0.08
Lansoprazole 30 mg kg^−1^ b.w.	33.23^b,c^ ± 2.4	1.240^b^ ± 0.12

Different letters “a” to “d” in the column represents that values are significantly different when compared between ulcer induced with healthy control and GRAE/lansoprazole-treated groups. Range was provided by Duncan multiple test at *P* < .05. ^a^Less significant; ^b^Moderately significant; ^c^Very significant and ^d^Most significant.

**Table 4:** Toxicity studies with GRAE (*n* = 6) mean ± SD.

Parameters	Control	GRAE treated
Total protein	348^a^ ± 32.21	358.43^a^ ± 22.1
SGOT (U mg^−1^ protein)	18.34^a^ ± 1.55	16.86^a^ ± 1.64
SGPT (U mg^−1^ protein)	21.31^a^ ± 2.70	18.91^a^ ± 2.42
ALP (U mg^−1^ protein)	35.52^a^ ± 3.879	36.82^a^ ± 2.91
TBARS (nmol mg^−1^ protein)	0.166^a^ ± 0.08	0.148^a^ ± 0.09

SGPT, Serum glutamate pyruvate transaminase; SGOT, Serum glutamate oxaloacetate transaminase; ALP, Alkaline phosphatase. ^a^ *P* < .05 between control and GRAE-treated groups.

**Table 5:** Characteristic biochemical tests for *H. pylori.*

Tests	Results
Urease	+ve
Catalase	+ve
Oxidase	+ve
Gram staining	Gram negative
Motility	Motile
Colony characteristic	White mucilage type
Antibiotics	
Erythromycin	Resistant
Nalidixic acid	Resistant
Polymixin	Resistant
Penicillin	Resistant
Vancomycin	Resistant
Amoxicillin	Susceptible
Clarithromycin	Susceptible
Metronidazole	Susceptible

Helicobacter pylori obtained from endoscopic excision was subjected to various biochemical tests to confirm the identity of isolated bacteria as *H. pylori*. Gram staining, enzyme analysis and morphological analysis as well as antibiotic resistance/susceptibility were included in the tests for characterization of *H. pylori*.

**Table 6:** Relative percentage contribution of individual phenolic acids towards anti-oxidant, anti-*H. pylori* and H^+^, K^+^-ATPase inhibition.

GRAE	Phenolics (mg g^−1^)	AOX (% contribution)	PPI (% contribution)	*H pylori*-inhibition (% contribution)
Gallic acid	3.4	93	9	66
Cinnamic acid	3.8	1	88	30
Other phenolics	0.4	6	3	4

Gallic acid significantly contributed to anti-oxidant activity than cinnamic acid where as cinnamic acid contributed to H^+^, K^+^-ATPase inhibition.
